# Sulphuretted Hydrogen as a Product of Indigestion, and Its Action upon the System

**Published:** 1885-04

**Authors:** John A. McLaughlin


### Art. XIII.—SULPHURETTED HYDROGEN AS A PRODUCT OF INDIGESTION, AND ITS ACTION UPON THE SYSTEM.

BY JOHN A. MCLAUGHLIN, D.V.S.

For a long time the subject of indigestion has been of more than ordinary interest to me ; in fact, I may say it constitutes a hobby with me ; a hobby which I am by no means ashamed of ; a hobby which, if a few more veterinarians would indulge in, even though they should become enthusiastic on it to the verge or even the lowest depths of fanaticism, would, when firmly convinced, be of decided benefit to our profession.

When I speak of indigestion, I include not only those disturbances which affect the alimentary canal, but the functional derangements of the liver as well, either of which may be an outcome of the other, though not necessarily so.

The process of digestion and the functions of the liver are absorbing physiological studies in themselves, but if we will but go beyond these studies in the abstract, and see how many diseases are but the outcome of a disordered digestion, the profession will not marvel at my enthusiasm on the subject, but may, instead, permit me to wonder at the many crude conceptions of diseases which many veterinarians, who write for the American veterinary journals, entertain; nor do I allude only to the poor, uneducated, diplomaless country practitioner, but I regret to include many of those holding diplomas, and priding themselves on their *Alma Maters*, and even being in charge of "chairs" in our "Veterinary Colleges."

In an article* which appeared in the May number, 1884, of the *U. S. Veterinary Journal*, I maintained that Azoturia was due primarily to a functional disturbance in the liver; I still, after several months, entertain similar views, and my treatment of it, and similar disorders, which has been mainly directed at the liver, has proved extremely gratifying. But it is not of Azoturia I would speak, though I wish to call to mind the paralysis which is found in that disorder, and a similar symptom in the present case, supposed to be due to an entirely different cause.

About January 30th, a bull bitch about nine or ten years old, was brought to me for treatment; at that time she was suffering from a severe attack of pneumonia, and had been suffering so for about ten days previous; subsequently she developed convulsions, which resulted in a slight paralysis, if I can use that term to designate some loss of power, behind. No one, I believe, will accuse pneumonia of producing the convulsions, though I can readily understand that pneumonia or any sickness is not conducive to an improvement of a disabled digestion; but the fact that the bitch had been covered some two weeks before I saw her, might have had something to do with the dyspepsia and liver trouble from which the animal

---

* Functional Derangements of the Liver and Azoturia.

was suffering, both evidenced by a diarrhœa, the color of the fæces, which were a decided though dirty yellow, and the odor emanating therefrom was something terrific; it surpassed in stench the alleged poor but nevertheless powerful specimen of *sulphuretted hydrogen*, which the professor in chemistry brought to the college one day as an illustration of what he described as a stinking stink. Twenty-four hours afterwards, though the fæces was well covered with ashes and chloride of lime, it caused emesis in a healthy man twenty feet away, and its aroma hung round the locality for a week.

For several days the bitch remained in a dead, heavy slumber, not easily awakened, and recognizing no one. On being stirred up, she would move around for a short time, and if permitted, would then go back to her box, to sleep, or willing to sleep in any place, preferring, however, to lie near the fire, for she was cold, and shivered even in a warm room.

She had her first convulsion on February 2d, about 2.30 A. M., just as I was about to retire, after returning from a call. The second occurred at 11.35 A. M.; the third at 3 P. M., and the fourth and last at 5 P. M. The symptoms of the convulsion were opisthotonos, the head being forced backward until it nearly touched the back, and a struggling with the forward limbs. As the convulsion was an utter surprise to me, and the symptoms were those which might occur in a case of asphyxia, I attempted to loosen the collar; but finding it all right, I immediately opened the door, and permitted the cool air to play upon her. It lasted about one minute. As recovery set in her inspirations became full and deep, and when fully over, she seemed decidedly more astonished and dumbfounded than I was, looking at me with an expression on her face which plainly wanted to know what the trouble was. She was extremely affectionate, and considerably damaged a good suit of clothes which adorned my person with her saliva, which accumulated in her mouth and on her lips during the spell. She whined for a considerable length of time after, but this was in all probability due to her surprise, as her whining was not repeated after her subsequent attacks; otherwise the symptoms were the same, except that she did not recover from the latter ones quite so easily and rapidly, though the difference was but a few seconds. In one of these convulsions I took particular pains to see if she retained consciousness, and am sure she did, for she seemed to look at me with open, grateful eyes, for my assistance in keeping her head from being forced backward. While assisting her head in this manner during the two last convulsions, the head made four or five lateral movements previous to the subsidence of the convulsion. After her three last fits she would fall into her dead, heavy sleep much more quickly than she did after her first. Each fit was rendering her weaker and weaker.

Now, let us arrive, if possible, at a cause of this intense disturbance of the system which resulted in such exaggerated symptoms. Quoting from my own article on "Functional Derangements of the Liver and Azoturia": "It seems not at all unlikely," wrote Dr. Lander Burton, F.R.S., in 1880, "that the liver has got another function besides those usually assigned to it, viz., that of preventing the digestive ferments from reaching the general circulation so as to act upon the tissues." Was it this function of the liver that was affected in this case? I certainly believe it was, and that the convulsions were, in all probability, due to the absorbtion of some poison from the alimentary canal.

It would appear, also, that peptones, when escaping into the general circulation, are depressent agents. Prof. Albertoni has found that peptones have a most remarkable action upon the blood, completely destroying the coagulability in dogs, while they have little power in this respect over the blood of rabbits and sheep. He and Dr. Schmidt-Muhlheim independently made the discovery that peptones prevented the coagulation of the blood in dogs, and the latter, under Ludwig's direction, has also investigated their action upon the circulation. He finds that when injected into the veins they greatly depress the circulation, so that the blood pressure falls considerably, and when the quantity injected is large, they produce a separate condition, complete arrest of the secretion by the kidneys, convulsion and death. From these experiments it is evident that the normal products of digestion are poisons of no inconsiderable power, and that if they reach the general circulation in large quantities they may produce very alarming, if not dangerous, symptoms. "It would appear that in certain conditions, as a consequence of disturbance in the working of the liver, the organism is not only ill-fed from the absence of normal products of assimilation, but is actually poisoned by the presence of products which are positively harmful." (Fothergill.)

It was not, however, the normal products of digestion that were absorbed in this case, at least not entirely, for though there were convulsions, there was no arrest of the secretion of the kidneys, for the bitch urinated immediately after two, if not three, of her fits, and never experienced any trouble in that way, though I can readily understand the symptoms produced by peptones varying greatly in different cases. But there is another factor in this case which I have mentioned, and that is the formation of a powerful disagreeable gas, which was no doubt sulphuretted hydrogen, the formation of which gas in the intestine Fothergill mentions, and suggests the probability of its absorption into the circulation. In this case I believe it was the absorption of sulphuretted hydrogen which was the cause of the convulsions from which the animal suffered. Sulphuretted hydrogen is a powerful poison, and acts on the system in a similar manner to hydrocyanic acid; and the action of this latter powerful poison is thus described by Finley Dunn: "One to four drops placed on the tongue, or within the eyelids of dogs, cats, rabbits, or such small animals, begin to operate in ten to thirty seconds; three or four rapid, labored inspirations, a hurried convulsive expiration, and a *general tetanic seizure* precede death. When life is prolonged beyond a few minutes the general sedative effect of the drug is demonstrated; there is paralysis of mobility, sensibility, and reflex irritability, muscular tremblings and convulsions;" other symptoms are a "profuse salivation, dilatation of the pupil, impaired power of voluntary movement." "According to Dr. Mayer, it acts by paralyzing the heart, being conveyed into the blood, and operating directly on the organ. The pulse is sometimes quickened, at other times reduced in frequency. Occasionally salivation and ulceration of the mouth are produced. When not immediately fatal, the symptoms produced are sudden loss of sense, trismus, difficulty and rattling respiration, coldness of the extremities, immobility, and sometimes contraction of the pupils, convulsions, etc." (Dispensatory). In the case of the bitch there was a decided action on the heart, for although I did not examine it at the time, on the

7th of February, five days later, the pulse was about 78, irregular and intermittent, there was a slight paralysis which has not left up to the present date; it is not entirely confined to the hind extremities, for she seems drawn slightly to one side, which is suspicious, at least, of some trouble in the medulla; there was a check of the breathing at first, and deep drawn inspirations later in the attacks, and some salivation, all of which are symptoms of poison by sulphuretted hydrogen or hydrocyanic acid, the difference in the symptoms being accounted for by the difference in those, which would naturally be expected from an experiment where the poison would be introduced in its pure state into the blood, and its absorption from the alimentary canal, in conjunction with other mal-products, due to a disordered digestion.

The case is of decided interest, and even though a theory is not really correct, it is well to have some basis from which to judge of the proper medicinal remedies to use. In this instance I concluded that the liver was at fault—decidedly at fault—and if the result justifies the treatment (of which I am sadly convinced it does not), I will say that the treatment in this case was perfectly correct, as she never took another fit after the first dose, although she remained very logy and sleepy for several days; for the last three days she has been lively enough, evidently revelling in her usual healthy proclivities for hunting and catching rats.

I have tried to find some history of the animal, and am informed that she never had a fit before, and the astonished expression on her face after the first one, certainly leads me to believe that she was not very familiar with such escapades; but being a remarkably good ratter, she was used by the neighborhood to show her excellence in that delectable pastime, and as a reward for her prowess, and the amusement she afforded the on-lookers, she was doubtless treated in the best manner by those who employed her services.

